# J147 Reduces tPA-Induced Brain Hemorrhage in Acute Experimental Stroke in Rats

**DOI:** 10.3389/fneur.2022.821082

**Published:** 2022-03-02

**Authors:** Rong Jin, Min Wang, Wei Zhong, Charles R. Kissinger, J. Ernest Villafranca, Guohong Li

**Affiliations:** ^1^Department of Neurosurgery and Neuroscience Institute, Penn State Hershey Medical Center, Hershey, PA, United States; ^2^Abrexa Pharmaceticals, Inc., San Diego, CA, United States; ^3^Department of Neurosurgery, Penn State Hershey Medical Center, Hershey, PA, United States

**Keywords:** ischemic stroke, J147, tPA, hemorrhage, cerebroprotection

## Abstract

**Background and purpose:**

J147, a novel neurotrophic compound, was originally developed to treat aging-associated neurological diseases. Based on the broad spectrum of cytoprotective effects exhibited by this compound, we investigated whether J147 has cerebroprotection for acute ischemic stroke and whether it can enhance the effectiveness of thrombolytic therapy with tissue plasminogen activator (tPA).

**Methods:**

Rats were subjected to transient occlusion of the middle cerebral artery (tMCAO) by insertion of an intraluminal suture or embolic middle cerebral artery occlusion (eMCAO), and treated intravenously with J147 alone or in combination with tPA.

**Results:**

We found that J147 treatment significantly reduced infarct volume when administered at 2 h after stroke onset in the tMCAO model, but had no effect in eMCAO without tPA. However, combination treatment with J147 plus tPA at 4 h after stroke onset significantly reduced infarct volume and neurological deficits at 72 h after stroke compared with saline or tPA alone groups in the eMCAO model. Importantly, the combination treatment significantly reduced delayed tPA-associated brain hemorrhage and secondary microvascular thrombosis. These protective effects were associated with J147-mediated inhibition of matrix metalloproteinase-9 (MMP9), 15-lipoxygenase-1, and plasminogen activator inhibitor (PAI) expression in the ischemic hemispheres (predominantly in ischemic cerebral endothelium). Moreover, the combination treatment significantly reduced circulating platelet activation and platelet-leukocyte aggregation compared with saline or tPA alone groups at 24 h after stroke, which might also contribute to reduced microvascular thrombosis and neuroinflammation (as demonstrated by reduced neutrophil brain infiltration and microglial activation).

**Conclusion:**

Our results demonstrate that J147 treatment alone exerts cerebral cytoprotective effects in a suture model of acute ischemic stroke, while in an embolic stroke model co-administration of J147 with tPA reduces delayed tPA-induced intracerebral hemorrhage and confers cerebroprotection. These findings suggest that J147-tPA combination therapy could be a promising approach to improving the treatment of ischemic stroke.

## Introduction

Stroke is a leading cause of morbidity and mortality worldwide (1). About 87% of all strokes are ischemic strokes, in which blood flow to the brain is blocked (1, 2). Since approval in 1996, intravenous administration of recombinant tissue-type plasminogen activator (rtPA) is the only Food and Drug Administration (FDA)—approved medication indicated for the treatment of patients with acute ischemic stroke (3). However, only 3–5% of all the patients with acute ischemic stroke in the United States receive IV tPA due to the extremely short timeframe for thrombolysis and the increased risks of intracranial hemorrhage with delayed tPA treatment (3, 4). It is well-known that IV tPA beyond 3 h of stroke onset significantly increases cerebral bleeding although it may be safe and effective in selected patients up to 4.5 h after symptom onset (5, 6). Thus, there is an urgent need for developing adjuvant therapeutic approaches that extend the therapeutic time window of tPA and reduce intracerebral hemorrhagic transformation due to delayed tPA treatment, making the thrombolytic therapy accessible to more patients with acute stroke.

**Figure 1:**
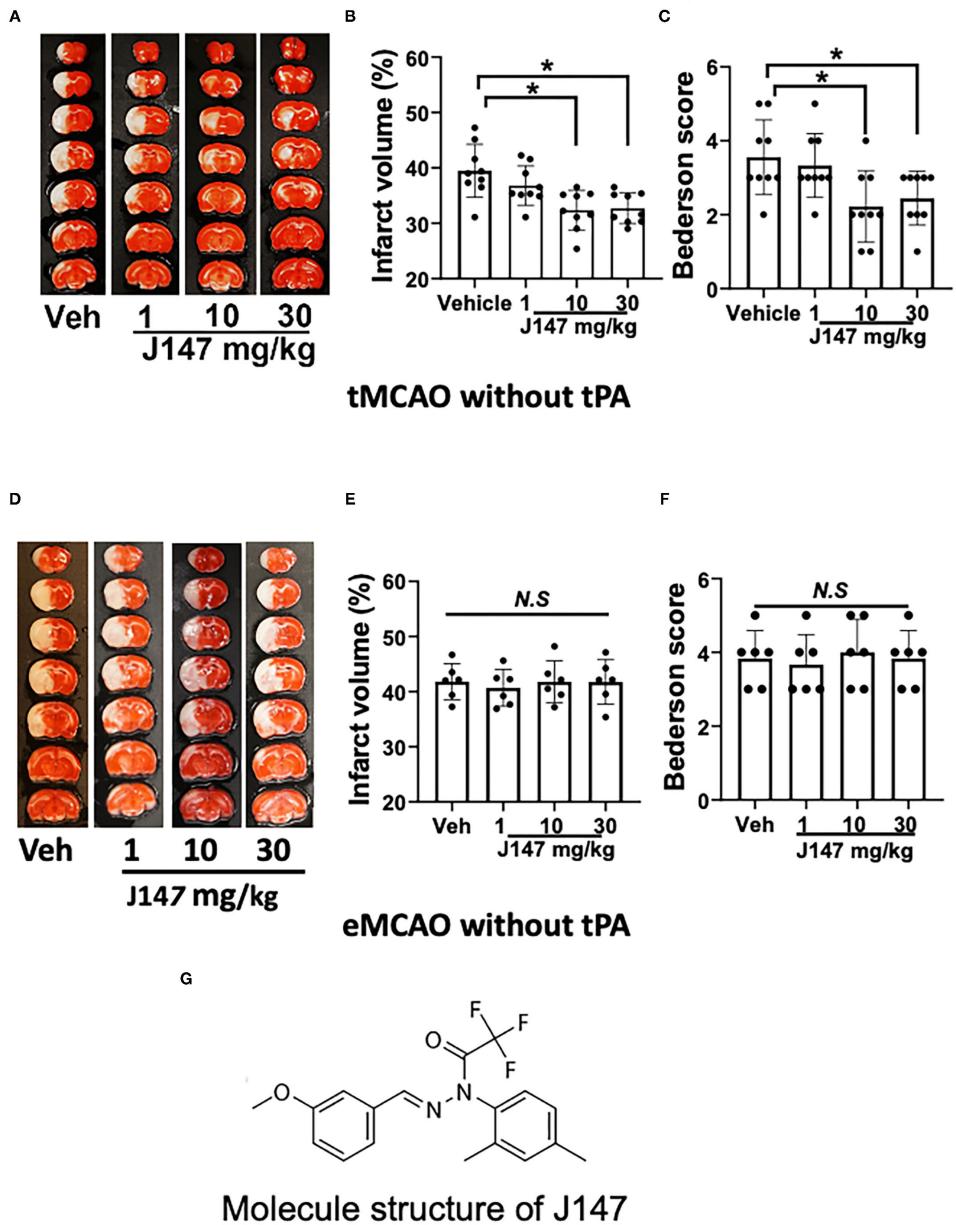
J147 treatment reduces brain damage after tMCAO but not in eMCAO. **(A)** Representative TTC staining; **(B)** infarct volume; **(C)** Bederson score; *n* = 9 per group [**(A–C)**; animals were subjected to 2 h tMCAO followed by reperfusion]; **(D–F)** TTC staining, infarct volume, and Bederson score. *n* = 6 per group [**(D–F)**; animals were subjected to eMCAO]. One-way ANOVA followed by the Bonferroni *post-hoc* test with selected multiple comparisons was used to compare infarct volume. Non-parametric functional scores were compared by Kruskal–Wallis test with *post-hoc* Dunn corrections. *p < 0.05. n.s., not significant. **(G)** Molecule structure of J147.

There has been a longstanding interest in the use of neuroprotective agents in stroke treatment, but clinical results have been consistently disappointing (7). Because of the complex pathogenesis of ischemic stroke and lack of success with single-target neuroprotective agents, there has been growing interest in therapeutic agents with pleiotropic effects. J147[systematicname: N-(2,4-dimethylphenyl)-2,2,2-trifluoro-N′-[(E)-(3-methoxyphenyl) methylene]acetohydrazide] is small molecule compound originally developed for treating neurodegenerative conditions associated with aging. The molecular structure of J147 is shown in Figure 1G. It was designed to mimic curcumin neuroprotective activity, but curcumin and J147 are not chemically related (8–10). Although curcumin has many proven beneficial effects on brain health, the clinical application of curcumin is limited by its poor bioavailability and low blood-brain barrier (BBB) penetration (11–13), both of which interfere with its therapeutic efficacy. In contrast, J147 shows good bioavailability, strong *in-vivo* neuroprotection, and can easily cross the BBB (10, 14, 15). J147 has many biological effects, including reducing inflammation and oxidative stress (8, 16), modulating Ca^2+^ metabolism (17), protecting the blood–brain barrier (BBB) permeability homeostasis (18), improving brain vascular function (18), and promoting amyloid-beta (Aβ) metabolism (10). It has been shown to have neuroprotective and neurotrophic effects in mouse models of Alzheimer's disease and accelerated aging, with no recorded side effects (10, 19, 20). J147 can also exert a protective role against glutamate excitotoxicity *in vitro* and *in vivo* (21). The neuroprotective and neurotrophic effects of J147 are correlated with its induction of brain-derived neurotrophic factor (BDNF) and nerve growth factor (NGF), both of which are known to have a potent ability to attenuate neuronal injury and repair brain damage (22–24). The α-F1 subunit of mitochondrial ATP synthase (ATP5A), a central player in Ca^2+^ metabolism, is identified as a target of J147, by which J147 can maintain intracellular Ca^2+^ homeostasis through regulation of store-operated calcium entry (SOCE), thereby reducing cell death during neurotoxicity (17). In addition, previous studies have shown that J147 can also induce 5-HT1A receptor expression and stimulate its-related signaling pathway (25), acting as a serotonergic target for neuroprotection in cerebral ischemia (26). Based on this unique spectrum of cerebral cytoprotective effects, and limited side effects, we identified J147 as a promising cerebroprotective agent for combined therapy with IV tPA. In this study, we aimed to investigate the cerebral cytoprotective effect of J147 in ischemic stroke and determine whether and how J147 extends the therapeutic time window for IV tPA in a rat model of embolic stroke.

## Materials and Methods

Data supporting the findings of this study are available from the corresponding author on reasonable request. This study adheres to the American Heart Association Journals' implementation of the Transparency and Openness Promotion guidelines. All experimental protocols were approved by the Institutional Animal Care and Use Committee at the Penn State University College of Medicine. A detailed Methods section is provided in the online-only Supplementary Materials. The Checklist of Methodological and Reporting Aspects is shown in Supplementary Table I.

### Suture Transient MCAO and Embolic MCAO Models

Adult male Wistar rats (weighing 280–320 g, purchased from Charles River Laboratories, Wilmington, Massachusetts, USA) were subjected to suture transient or embolic middle cerebral artery occlusion (MCAO) according to the standard operating procedure (27) (please see Supplementary Methods for detail). For the suture transient MCAO model (tMCAO), animals underwent 2 h of ischemic stroke using silicone rubber-coated monofilaments (503956PK5RE, Doccol, Sharon, Massachusetts, USA). For embolic MCAO models (eMCAO), a single 4 cm fibrin-rich clot was placed in the origin of the right middle cerebral artery (MCA) *via* a modified PE-50 tube (0.3-mm outer diameter). Regional cerebral blood flow (rCBF) in the MCA territory (2 mm posterior and 5 mm lateral to the bregma on the right parietal skull) was monitored with Laser Doppler flowmetry (MSP300XP; ADInstruments Incorporation). The rCBF reduction of animals to 25% or less of baseline level was included in the study.

### Experimental Protocols

To test the dose-response of J147, rats were subjected to tMCAO or eMCAO and randomly assigned into the following 4 groups: vehicle [DMSO/PEG200/saline (5/70/25%)] and varied doses of J147 including 1, 10, and 30 mg/kg. J147 or equal volume of vehicle was administrated *via* femoral vein at 2 h after ischemia onset using a syringe infusion pump (Harvard Apparatus, Holliston, MA, USA) for 2 min. Rats were euthanized at 72 h after stroke. Based on the dose-response assessment, the optimal dose of J147 (10 mg/kg) was used in the following studies. To test the J147-tPA combination therapy, eMCAO rats were randomly assigned to the following 3 groups: treated with vehicle plus saline (indicated as a saline group), vehicle plus rtPA (10 mg/kg) (as rtPA group), and J147 plus tPA (as combination therapy group) at 4 h after the onset of ischemia. For the combination treatment, the J147 or vehicle was administrated *via* femoral vein for 2 min, followed by human recombinant tPA (Alteplase; Genentech, Inc, San Francisco, California, USA) with 10% as a bolus and 90% as a 30-min infusion using a syringe infusion pump (Harvard Apparatus, Holliston, Massachusetts, USA). All the animals were euthanized 3 days after stroke with CO_2_. The modified Bederson score, used to determine global neurological function, was performed by a blinded investigator before and at 72 h after stroke, as we described previously (28, 29). For randomization, the web tool www.randomizer.org was used. Animals were randomly assigned to each group *via* random numbers generated on an Excel spreadsheet. The number of animals used in each experimental group and the total number of animals used in this study are summarized in Supplementary Table II.

### Infarct Volume and Intracerebral Hemorrhage

The infarct volume was measured in TTC-stained coronal sections at 72 h after stroke. Then, the TTC-stained sections were homogenized and the hemoglobin levels were assessed by a spectrophotometric assay using Drabkin reagent (Sigma-Aldrich), as we previously described (28, 29).

### Immunohistochemistry

Immunostaining was performed as we described previously (28, 29). The primary antibodies: Iba-1 (1:200, Wako, VA, USA), myeloperoxidase (MPO) (1:200, Abcam, MA, USA), matrix metalloproteinase-9 (MMP-9) (1:100, R&D, MN, USA), endothelial barrier antigen (EBA) (a marker for endothelium in the vessels, 1:500, Covance, NJ, USA), fibrinogen (1:200, Dako, CA, USA), thrombocyte (1:200, Lifespan, WA, USA), and plasminogen activator inhibitor-1 (PAI-1) (1:200, Novus Biologicals, CO, USA) were used. Isotype controls were used as negative controls to help differentiate non-specific background signals from specific antibody signals. Serial frozen coronal sections (15 μm thick) were collected from 0.4 to 1.4 mm posterior to bregma. Every 10th coronal section for a total of five sections was used for immunohistochemistry. To maintain consistency within and the between animal groups, two predefined fields within the cortical ischemic boundary zone were selected as the region of interest (ROI) as shown in **Figure 3**. The number of positive cells or microvessels in each ROI was counted, and the data were expressed as mean ± SD of positive cells/microvessels per mm^2^. All immunostaining data were analyzed by an investigator blinded to experimental groups using Image Pro plus software (version 5.1, Media Cybernetics Incorporation, MD, USA).

### Gelatin Zymography and Western Blot

Two assays were performed as described previously (30, 31). Protein extracts were obtained from the cerebral cortices (bregma +1 to −2 mm). MMP-9 activity in brain homogenates was determined by gelatin zymography. For Western blot, brain samples were homogenized in cold radioimmunoprecipitation assay buffer containing protease inhibitor cocktail. Protein samples (40 μg protein each lane) were separated by sodium dodecyl sulfate-polyacrylamide gel electrophoresis and transferred to polyvinylidene difluoride membrane. Anti-15 lipoxygenase 1 (ab244205, 1:1,000) was used. Then, the membrane was stripped and re-probed with beta-action (A2066, 1:500) antibody. Ponceau S staining and beta-actin staining were used for loading control.

### Platelet Activation and Platelet-Leukocyte Interactions

Whole blood (500 μl) was drawn into a heparinized capillary tube *via* the retro-orbital plexus at 24 h after stroke. The samples were analyzed on the BD Accuri™ C6 flow cytometer, as we previously described (please see Supplementary Materials for detail) (28). Isotype-matched control antibodies were used to differentiate non-specific background signals from specific antibody signals. Blood samples were run on a BD Accuri™ C6 Flow Cytometry and data were analyzed by FlowJo software.

### Pharmacokinetic Study

#### Sample Preparation

The concentration levels of J147 in rat plasma and brain were evaluated using the high-performance liquid chromatography tandem mass spectrometry (HPLC-MS/MS) method, and dexamethasone (50 ng/ml final concentration) was used as an internal standard. Normal rats received a single IV dose of 10 mg/kg J147. Whole blood and brain samples were collected at indicated time points and prepared for analysis (please see Supplementary Materials for detail).

#### HPLC-MS/MS Analysis of J147

J147 in plasma and brain homogenate was analyzed using an AB API 5500 mass spectrometry coupled with Shimadzu HPLC separation system and all peaks were integrated and quantified using Analyst 1.7.1 and 1.6.2 software (please see Supplementary Materials for detail).

### Statistical Analysis

Data are expressed as mean ± SE of the mean. GraphPad Prism 8 software package was used for statistical analysis. The normality of data was assessed with the D'Agostino-Pearson omnibus test. For normally distributed variables, one-way ANOVA followed by the Bonferroni *post-hoc* test was used to assess differences between groups. If only 2 groups were compared, an unpaired, 2-tailed Student's *t*-test, was applied. The Kruskal–Wallis and Mann–Whitney *U* tests were used to explore differences between groups in non-normally distributed variables. Non-parametric functional outcome scores were compared by the Kruskal–Wallis test with *post-hoc* Dunn corrections. Sample size calculation (power = 0.8, α = 0.05) was performed using an online calculator (http://www.lasec.cuhk.edu.hk/sample-size-calculation.html). Based on our previous infarct volume data using the same stroke model (18, 19) estimated 6 animals per group would be required to detect an infarct equivalent to 25% of the uninjured hemisphere. For comparison of survival data, the log-rank test was used. *p* < 0.05 was considered statistically significant.

## Results

### J147 Treatment Improves Ischemic Outcomes After tMCAO but Not in eMCAO Without tPA

Our results show that J147 treatment decreased infarct volume in a dose-dependent manner in tMCAO. Infarct volumes were significantly reduced in rats treated with J147 at a dose of 10–30 mg/kg (*p* < 0.05), whereas a lower dose of 1 mg/kg J147 had no significant effect (*p* > 0.05) compared to the vehicle-treated control group (Figures 1A,B). Importantly, the smaller infarct volumes translated into a better neurological outcome. Animals treated with J147 (10 or 30 mg/kg) showed significant improvement in Bederson score compared to vehicle controls (Figure 1C). In addition, there was no significant difference between 10 and 30 mg/kg of J147-treated groups in brain damage and neurological deficits. Based on these data, J147 at the dose of 10 mg/kg was used for further experiments. We also evaluated the neuroprotective effects of J147 in rats subjected to eMCAO. The results show that infarct volume and neurological deficits did not differ significantly between vehicle- and J147-treatment groups (Figures 1D–F). This is most likely because reperfusion is not established without tPA and J147 does not have sufficient access to the ischemic brain to achieve therapeutic concentration and produce the desired neuroprotective effects. While J147 crosses the BBB readily, blood must reach affected areas for J147 to be effective there.

### J147 Plus tPA Combination Therapy Attenuates tPA-Associated Hemorrhage and Alleviates Brain Damage After eMCAO

**Figure 2:**
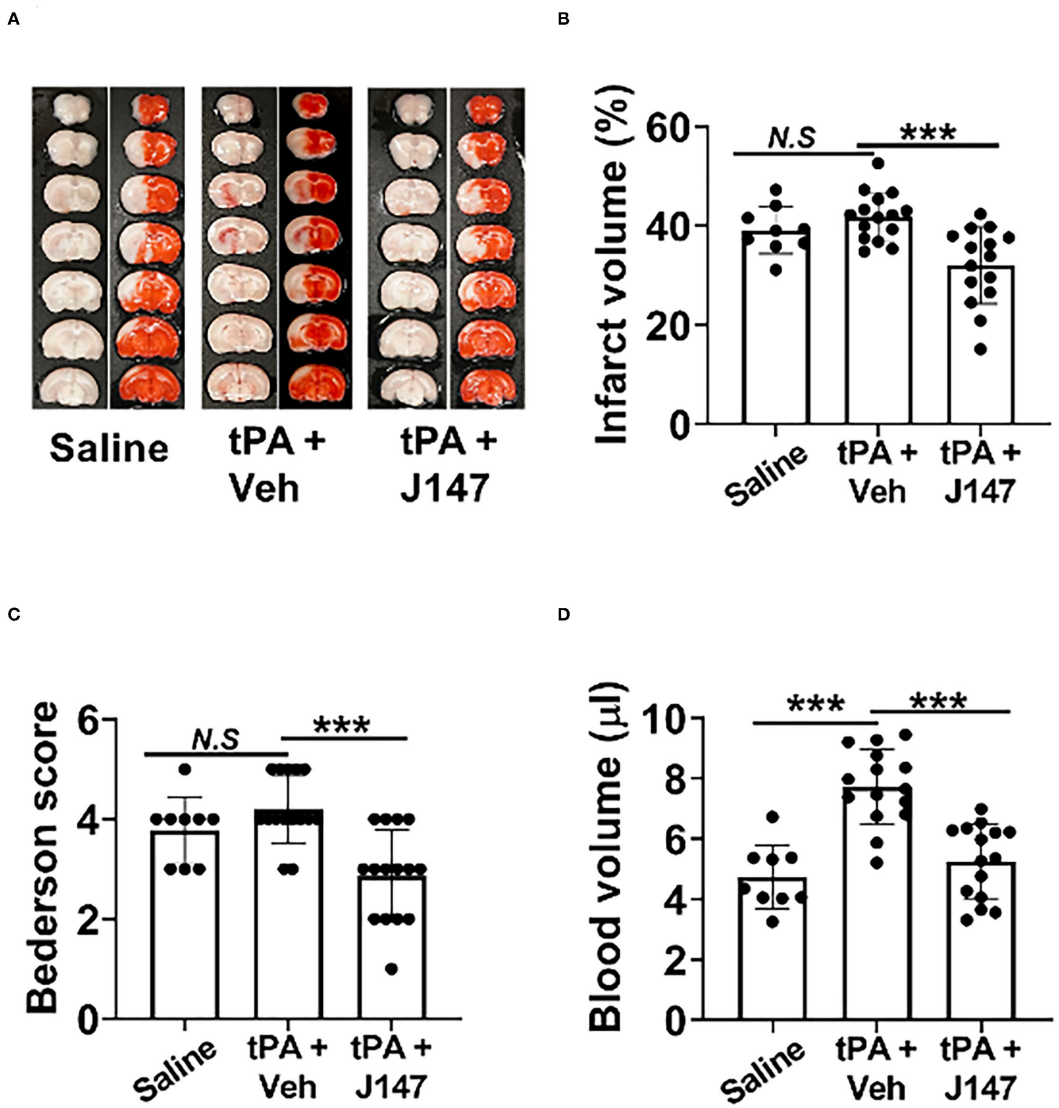
J147 plus tPA combination therapy reduces the risk of brain hemorrhage and ameliorates acute stroke injury. **(A)** Representative images of unstained coronal sections (red color: intracerebral hemorrhage) and TTC-stained sections in the indicated groups. **(B,C)** Quantitative analysis of infarct volume **(B)** and hemorrhage volume **(C)**. **(D)** Bederson score of the indicated groups 72 h after stroke. Animals were treated with rtPA (10 mg/kg) or a combination of tPA plus J147 at 4 h after the onset of ischemia. *n* = 9–15 rats per eMCAO group. ***p < 0.001.

Intracerebral hemorrhage (ICH) and ischemic stroke outcomes were assessed on day 3 after stroke. As expected, IV tPA at 4 h did not affect infarct volume but increased ICH (Figures 2A,B,D). However, the combination therapy of tPA + J147 at 4 h not only reduced the tPA-induced ICH (Figures 2A,D) but also significantly reduced infarct volume (Figures 2A,B) and improved neurological function (Figure 2C). The mortality rate in saline and rtPA groups were 25% (3 of 12 rats) and 31.38% (7 of 22 rats), respectively. The mortality rate was reduced to 15.4% (2 of 17 rats) in the combination, tPA + J147, treatment group.

### J147 Treatment Alleviates Delayed tPA-Enhanced Neuroinflammation

**Figure 3:**
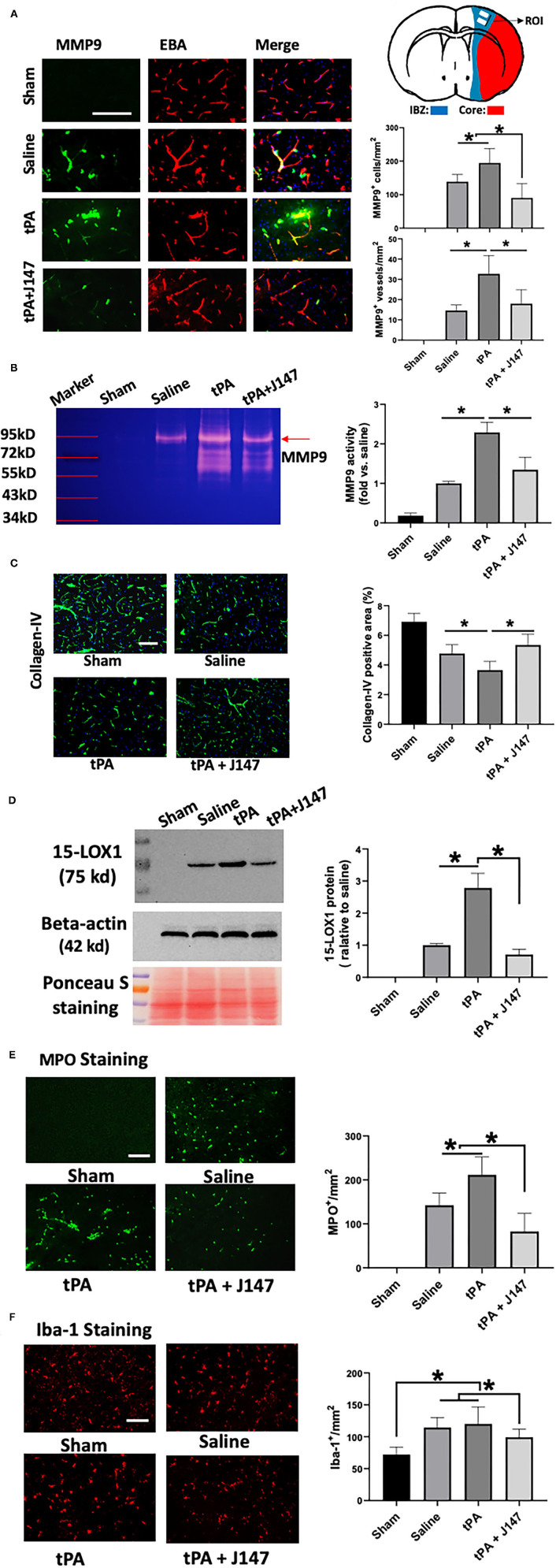
J147 treatment reduces ischemia-induced and delayed tPA-enhanced neuroinflammation. **(A)** Representative images of double immunofluorescence staining for MMP-9 with the endothelial barrier antigen (EBA) in the indicated groups. Quantitative analysis showing the combination treatment significantly inhibits MMP9 expression in both individual cells and microvessels compared to vehicle or rtPA alone groups. Top right: Schematic diagram showing the region of interest (ROI) for IHC. **(B)** Gelatin zymography. **(C)** Collagen-IV staining. **(D)** Western blot analysis of the expression of 15-LOX-1 after stroke. Ponceau s staining as the loading control. Data are normalized to beta-actin and expressed as fold change relative to the saline-treated group. **(E,F)** Brain neutrophil infiltration **(E)** and microglia expansion **(F)** in the indicated groups. Bar = 100 um. *p < 0.05. *n* = 5 animals per group.

Matrix metalloproteinases, in particular MMP-9, play a critical role in the stroke-associated BBB disruption, hemorrhagic transformation, and neuroinflammation after stroke (31). Double immunohistochemistry was performed to determine MMP-9 expression in the brain at 24 h after stroke (Figure 3A). As expected, MMP-9 immunoreactivity was absent in the sham-operated rats and markedly increased in stroke animals. In the delayed tPA treatment group, it was further increased, which was present on both the individual cells and brain microvessels (marked by endothelial barrier antigen staining). However, combination treatment of tPA + J147 markedly decreased the MMP9 expression on both individual cells and microvessels. The MMP9 activity was also evaluated with gelatin-zymography. The results showed that MMP-9 activity was significantly increased after ischemia and further enhanced by delayed tPA, and these effects were profoundly inhibited by combination treatment of tPA + J147 and J147 (Figure 3B), with a substantial improvement of collagen-IV degradation after stroke (Figure 3C). In addition, evidence shows that 12/15-lipoxygenase (12/15-LOX, also known as 15-LOX-1), one of the key enzymes of the arachidonic acid cascade, is upregulated following ischemic stroke and contributes to both neuronal cell death and hemorrhagic transformation (32–34). Western blot results showed that the signal of 15-LOX-1 was absent in the sham-operated rats and markedly increased in stroke animals at 24 h after stroke. In the delayed tPA treatment group, it was further increased. However, combination treatment of tPA + J147 markedly decreased the 15-LOX-1 expression (Figure 3D). We also evaluated the neutrophil (MPO) infiltration and microglia (Iba-1) expansion in the brain, both of which are involved in the post-ischemic neuroinflammation. Immunostaining showed that MPO-positive cells were not detected in the sham-operated rats, but were significantly induced by stroke and further augmented by delayed tPA treatment (Figure 3E). The combination therapy markedly reduced neutrophil infiltration into the brain parenchyma post-stroke (Figure 3E). Furthermore, the number of the Iba-1-positive cells was significantly increased after stroke. However, the combination treatment of tPA + J147 significantly blocked the microglia expansion; in contrast, delayed tPA alone showed no significant effect on the expansion of microglia compared with the saline alone group (Figure 3F). The shape of most Iba-1 positive cells was transformed from a highly ramified into a less ramified or amoeboid cell shape in saline and tPA alone group, indicating that microglia has been activated. Importantly, it was significantly inhibited by combination treatment of tPA + J147.

### J147 Treatment Alleviates Delayed tPA-Enhanced Microvascular Thrombosis

**Figure 4:**
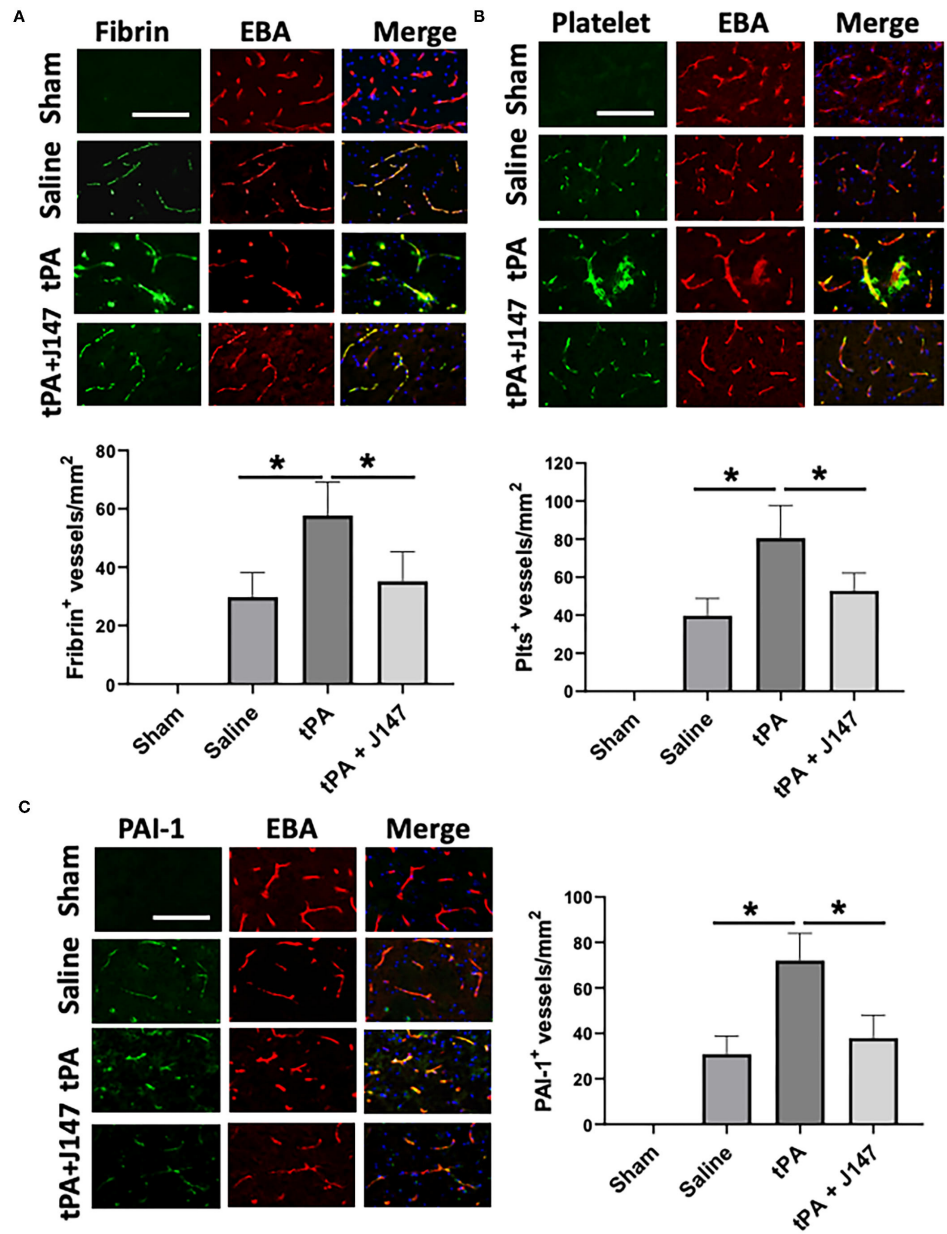
J147 treatment alleviates microvascular thrombosis and PAI-1 (plasminogen activator inhibitor-1) expression. **(A,B)** Representative images of double immunofluorescence staining showing fibrin [**(A)**, top) or thrombocyte [**(B)**, top) deposited in brain microvessels [marked by endothelial barrier antigen (EBA) staining]. The number of fibrin-positive [**(A)**, bottom] and thrombocyte-positive [**(B)**, bottom] vessels was counted as described in the Methods. **(C)** Representative images of double immunofluorescence staining for PAI-1 (green) with EBA (red) are indicated groups. The number of PAI-1–positive vessels was counted as described in the Methods. Bar = 100 um, *p < 0.05. *n* = 5 animals per group.

Intravascular fibrin/fibrinogen deposition and platelet accumulation substantially contribute to secondary microvascular thrombosis after ischemic stroke (35). Double immunofluorescence staining showed that intravascular fibrin/fibrinogen deposition (Figure 4A) and platelet accumulation (Figure 4B) were rarely detected in sham-operated rats and at a relatively low level detected in the saline-treated stroke animals, possibly because of low cerebral perfusion in downstream microvessels without tPA thrombolysis in this embolic stroke model. However, both the fibrin/fibrinogen and platelets deposited in downstream microvessels were markedly increased in stroke rats with delayed tPA, and these increases were significantly attenuated by combination treatment of tPA + J147 (Figures 4A,B). Evidence shows that local upregulation of PAI-1, a downstream mediator in the NF-κB cascade, in ischemic brain endothelium contributes to intravascular fibrin/fibrinogen deposition during ischemia/reperfusion-induced acute brain ischemic injury (36). Double IHC showed that combination treatment with J147 significantly reduced ischemia-induced and delayed tPA-enhanced PAI-1 expression in brain endothelial cells observed in the perilesional area of the cerebral cortex (Figure 4C).

### J147 Treatment Attenuates Circulating Platelet Activation and Platelet-Neutrophil Aggregates *in vivo*

**Figure 5:**
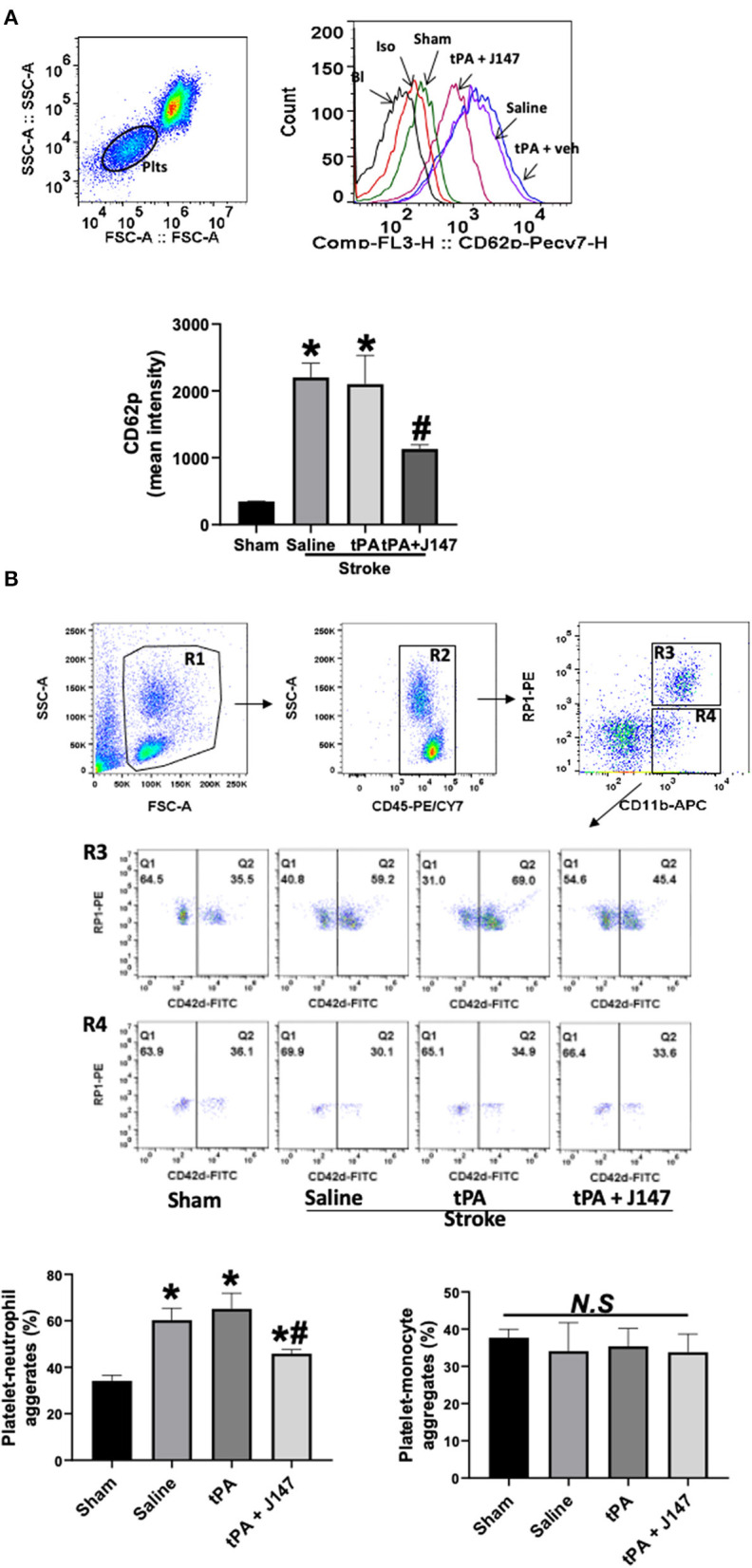
J147 treatment attenuates circulating platelet activation and platelet-neutrophil aggregates *in vivo*. **(A)** Flow cytometric measurement of platelet P-selectin expression in whole blood. Representative gating strategy of platelets in whole blood and histogram of P-selectin (CD62P) expression on platelets in the indicated groups. Data are expressed as mean fluorescence intensity. *p < 0.05 vs. sham group. # < 0.05 vs. saline or tPA groups. **(B)** Gating strategy for flow cytometric analysis of total leukocytes (R2, CD45 positive cells included in R1) in whole blood: neutrophils (N; R3) and monocytes (M; R4) were defined by their differential expression of CD11b and RP-1 (specific for rat granulocytes); and representative flow cytometric dot plots of platelet-neutrophil (CD42d^+^CD45^+^CD11b^+^PR1^+^) and –monocyte (CD42d^+^CD45^+^CD11b^+^PR1^−^) aggregates in the indicated groups. Fluorescein isothiocyanate (FITC)-labeled CD42d (glycoprotein V) mAb was used as a specific platelet marker to identify platelet neutrophil/monocyte complexes. The quantitative analysis showing the combination treatment significantly alleviates ischemia-induced and delayed tPA-enhanced circulating platelet-neutrophil aggregates, but not in platelet-monocyte aggregates. *n* = 5 rats per group. *p < 0.05 vs. sham; ^#^*p* < 0.05 vs. saline or tPA groups. N. S., not significant.

Elevated circulating platelet activation and platelet-leukocyte interaction contribute importantly to proinflammatory and thrombotic events during ischemia-reperfusion injury after stroke (37). We used flow cytometry to analyze platelet activation and platelet-leukocyte aggregates in whole blood at 24 h after ischemia onset. The increased platelet activation (determined by P-selectin expression) (Figure 5A) and platelet-granulocyte aggregates (Figure 5B) were found in the saline-treated stroke rats, but IV tPA had no additional effect, as we previously observed (28). However, combination treatment of tPA + J147 significantly reduced the ischemia-induced platelet P-selectin expression and platelet-granulocyte aggregates. There were no significant changes in the platelet-monocyte aggregates between groups.

### Pharmacokinetics and Tissue Distribution Study

**Figure 6:**
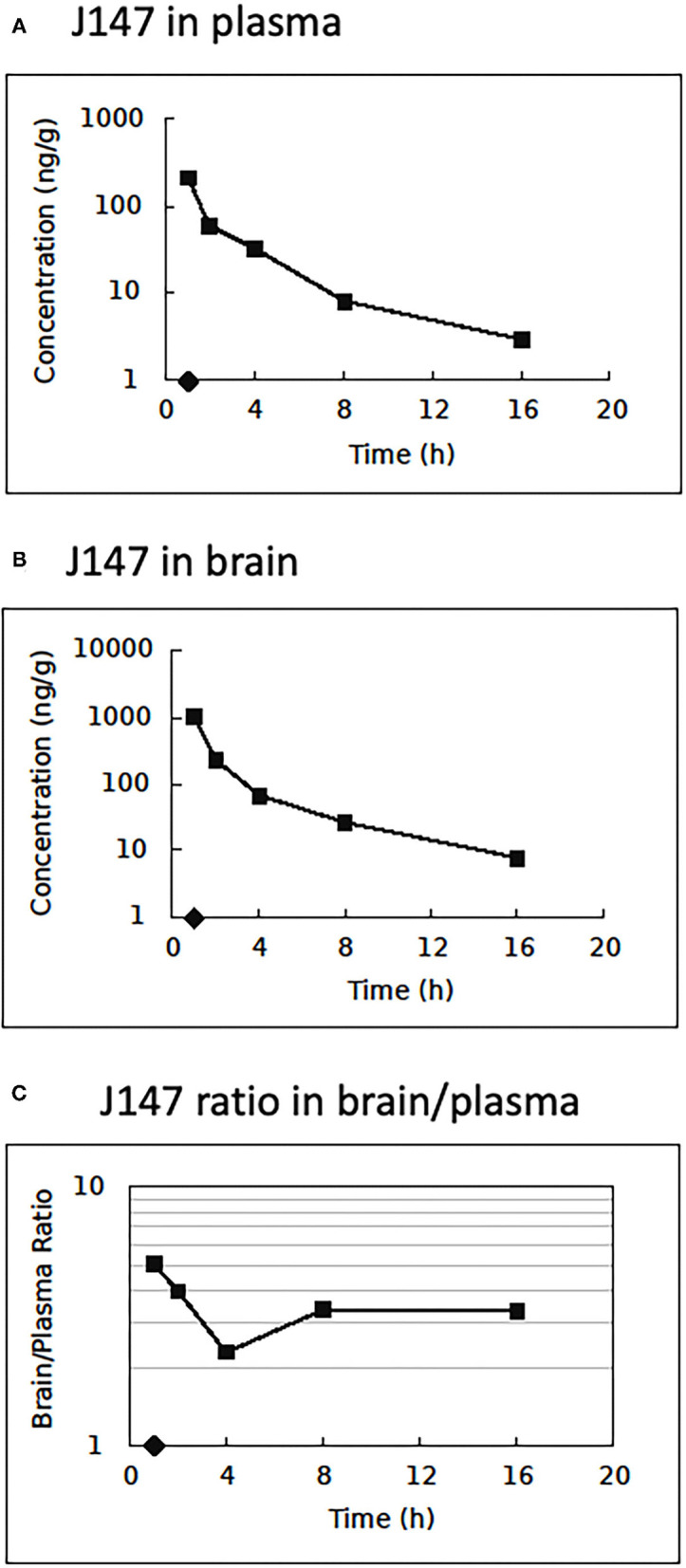
Pharmacokinetics and tissue distribution. Brain and plasma J147 concentrations after intravenous injection at 10.0 mg/kg. **(A)** Mean plasma concentration (*n* = 3/time point) vs. time after injection. **(B)** Mean brain concentration vs. time after injection. **(C)** The ratio of J147 distribution in brain and plasma vs. time.

The LC-MS/MS method was applied to quantify the drug distribution in rat plasma and brain tissue. Samples were obtained from rats after an IV administration of J147 solution [DMSO/PEG200/saline (5/70/25%)] at the dose of 10 mg/kg, the same formulation and optimal dose used in the tMCAO and eMCAO model experiments. The plasma and brain samples were collected at time points from 1 to 16 h. Figure 6 shows the mean plasma concentration-time curve, mean brain concentration-time curve, and ratio of J147 distribution in brain and plasma. The pharmacokinetic parameters of plasma are displayed in Supplementary Table III. The mean residence time was 1.72 h, and the *T*_1/2_ was 6.33 h.

## Discussion

This study demonstrates that the combination of J147 and tPA initiated at 4 h after embolic stroke substantially reduces brain damage and neurological deficits. The observed neuroprotective effects of combination therapy are likely attributed to reduced delayed tPA-induced ICH, reduced neuroinflammation, attenuated thrombosis formation in downstream microvessels, and reduced post-stroke platelet activation and platelet-leukocyte aggregation. These data suggest that J147 in combination with tPA may be a clinically feasible approach toward future attempts at combination stroke therapy.

J147 was originally developed for the use of treating neurodegenerative diseases associated with aging (8, 9, 18). It can easily cross the BBB into the brain and maintain biological stability (10, 14). J147 has several biological activities relevant to stroke, such as reducing oxidative stress and inflammation (8) (16), improving mitochondrial function (19, 20), promoting Aβ metabolism and reducing its levels in the brain (8, 10), increasing dendritic spine density and improving synaptic function (8, 38). J147 also has been reported to mitigate the decreased cell viability observed during acute neurotoxicity, which is most likely mediated through maintaining calcium homeostasis (17). At present, α-F1 subunit of mitochondrial ATP synthase (ATP5A), a central player in Ca^2+^ metabolism, and 5-HT1A receptor are identified as putative targets of J147. Engaging either of these exerts neuroprotective effects, either through maintaining calcium homeostasis or by inhibiting the firing activity of neurons in experimental models of brain injury (39). These findings prompted us to investigate the therapeutic potential of J147 in experimental stroke. In this study, treatment with J147 significantly reduced infarct volume when administered at 2 h after stroke onset in the model of tMCAO but was ineffective in the model of eMCAO. The different effects in the two models are consistent with a need for reperfusion to be established to allow therapeutic concentrations of J147 to reach the ischemic regions. The results raise the notion that the administration of J147 could act as an adjunctive treatment approach to address the limitations of tPA treatment and potentially expand the time window for ischemic stroke therapy. Thus, we tested the effects of combination therapy with J147 and tPA in the eMCAO model. The results show that the combination therapy significantly decreased the infarct volume and ameliorated neurological dysfunction. Encouragingly, the risks of intracranial hemorrhage with delayed tPA treatment were significantly decreased by the combination treatment with J147. The survival rate was also decreased in the combination treatment group compared to the saline and tPA alone group although it did not reach statistical significance because of the small animal numbers tested. Based on the above results, J147, as an adjunct to tPA, has the potential to reduce neuronal dysfunction and expand the thrombolytic time window in victims of acute ischemic stroke.

To investigate the mechanisms underlying the cerebroprotective effects of combination treatment with J147 and tPA, we first explored whether the combination therapy reduced ischemia-induced neuroinflammation and delayed tPA-associated hemorrhagic transformation. MMP-9, which is mainly derived from infiltrated neutrophils and inflammation-activated endothelial cells during ischemic stroke, plays a critical role in the delayed tPA-induced BBB disruption, hemorrhagic transformation, and neuroinflammation (40). Microglia activation is also established as a strong contributor to neuroinflammation in the pathogenesis of stroke (41). Our findings demonstrated that delayed tPA-treated animals had significantly upregulated MMP9 expression on both individual cells and microvessels compared with the vehicle group, as previously described (28). However, they were significantly inhibited by the combination therapy. The microglia expansion and activation were also markedly decreased in the combination treatment group compared with vehicle and tPA groups. In addition, our results also show that the combination therapy significantly decreased the expression of 15-LOX-1, which is a critical enzyme in the formation of lipid peroxidation, inducing inflammation and contributing to delayed tPA-related hemorrhagic transformation after ischemic stroke (32, 34).

We next hypothesized that combination treatment-initiated protective effects may occur through attenuated secondary microvascular thrombosis formation, thereby improving the success rate of reperfusion therapies and decreasing the neurological deficits after stroke. In this study, the results show that J147 exerts potent antithrombotic effects by reducing intravascular fibrin and platelet deposition, thereby reducing downstream microvascular thrombosis. The reduced downstream microvascular thrombosis was associated with a decrease in local endothelial-derived PAI-1 expression in the combination treatment group compared with tPA alone. Furthermore, we investigated whether J147 treatment could inhibit the stroke-induced circulating platelet activation and platelet-leukocyte aggregates, both of which link inflammatory and thromboembolic events in ischemic stroke (42). Our findings demonstrated that combination treatment with J147 and tPA profoundly inhibited circulating platelet activation and platelet-neutrophil aggregates elicited by MCAO. Taken together, our findings suggest that the microvascular protection by combination treatment with J147 and tPA probably involves a combined anti-inflammatory and antithrombotic mechanism.

J147 lowers levels of markers of oxidative stress in the rat hippocampus (8), and this is consistent with the observed anti-inflammatory effects of this compound following stroke. Recent evidence suggests that platelet activation in response to reactive oxygen species plays a major role in reperfusion injury (37). Platelets appear to play a complex, dual role in stroke etiology, however, also reducing hemorrhagic transformation and enhancing delayed repair, possibly by the direct release of stored BDNF (37). Increased BDNF expression is another notable effect of the J147 treatment. BDNF has diverse neurotrophic and neuroprotective effects, and there is extensive evidence suggesting BDNF plays a major role in the central nervous system (CNS) repair after stroke (43). The observed cerebroprotective effects of J147 after stroke might be explained by a favorable modulation of the inflammatory response and the balance between the damaging and protective activities of platelets and other components of the inflammatory response.

For J147 to succeed in the clinic, it must possess numerous pharmacological properties, including adequate pharmacokinetics (PK) and compatibility with tPA. The PK parameters in the rat after iv administration of the optimal therapeutic dose of 10 mg/kg show that J147 readily penetrates the CNS and that plasma and brain exposures exceed concentrations expected to be efficacious against the proposed biological targets. In addition, we have tested J147 *in vitro* for inhibition of tPA enzymatic activity. J147 does not inhibit tPA serine protease activity against plasminogen up to 10 uM (Supplementary Methods).

In summary, this study is the first to show the cerebral cytoprotective effects of J147 in acute ischemic stroke. The combination treatment with J147 significantly inhibited delayed tPA-enhanced hemorrhagic transformation, neuroinflammation, and downstream microvascular thrombosis. The results of this study indicate that these effects comprise the mechanisms of action of improved neurological functional outcomes of J147 after stroke and are suggestive of the meaningful clinical utility of J147.

## Perspectives

This study provides the first evidence that the combination treatment with J147 reduces delayed tPA-induced brain hemorrhage, neuroinflammation, and secondary microvascular thrombosis in a rat model of thromboembolic stroke, a widely used model to simulate reperfusion therapy *via* intravenous thrombolysis. Therapeutic potential with multiple mechanisms of action of J147 suggests that J147 represents a promising candidate drug for treating ischemic stroke, and thus merits further preclinical investigation and evaluation under comorbid conditions such as diabetes and hypertension. There are some limitations to this study. Mechanical thrombectomy is now the standard of care for acute ischemic stroke caused by large vessel occlusion. To reduce or prevent reperfusion injury, bridging therapy, i.e., combining intravenous thrombolysis and mechanical thrombectomy, has been increasingly applied in the clinic (44, 45), and the current American Heart Association/American Stroke Association guidelines also recommend this therapy (46). Our data have shown that J147 treatment alone when administered at 2 h after stroke significantly ameliorates acute stroke injury in a rat model of transient endovascular suture MCAO—a model used to simulate mechanical reperfusion therapy by endovascular thrombectomy. Whether J147 can improve bridging therapy warrants further study. Moreover, a pharmacokinetics and tissue distribution study was performed in normal control animals, which quantified the drug distribution in rat plasma and brain tissue and demonstrated that J147 readily crossed the blood-brain barrier. However, whether and to what extent the distribution of J147 from plasma to target brain tissue is compromised in animals subjected to transient or permanent MCA occlusion warrants further study.

## Data Availability Statement

The original contributions presented in the study are included in the article/Supplementary Material.

## Ethics Statement

The animal study was reviewed and approved by Institutional Animal Care and Use Committee at Penn State University College of Medicine.

## Author Contributions

RJ, MW, and WZ performed experiments and data analysis. RJ contributed to manuscript writing. JV and CK contributed to the pharmacokinetic study and manuscript revising. GL designed and supervised the study, wrote, and revised the manuscript. All authors contributed to the article and approved the submitted version.

## Funding

This is an industry-funded project (GL) supported by Abrexa Pharmaceticals, Inc. CA, USA and in part supported by the National Institutes of Health grants (NS089991 and NS119538 to GL).

## Conflict of Interest

The authors declare that this study received funding from Abrexa Pharmaceticals, Inc. The funder had the following involvement in the study: pharmacokinetic study and manuscript revising. JV and CK are employed by the Abrexa Pharmaceuticals Incorporation.

## Publisher's Note

All claims expressed in this article are solely those of the authors and do not necessarily represent those of their affiliated organizations, or those of the publisher, the editors and the reviewers. Any product that may be evaluated in this article, or claim that may be made by its manufacturer, is not guaranteed or endorsed by the publisher.
