# Supplementary material for: J147 Reduces tPA-Induced Brain Hemorrhage in Acute Experimental Stroke in Rats
Source: Front Neurol. 2022 Mar 2;13:821082. doi: 10.3389/fneur.2022.821082 (PMC8925862; doi:10.3389/fneur.2022.821082)
Supplement: Supplementary file 1 [file Presentation_1.PPTX]

## Slide 1
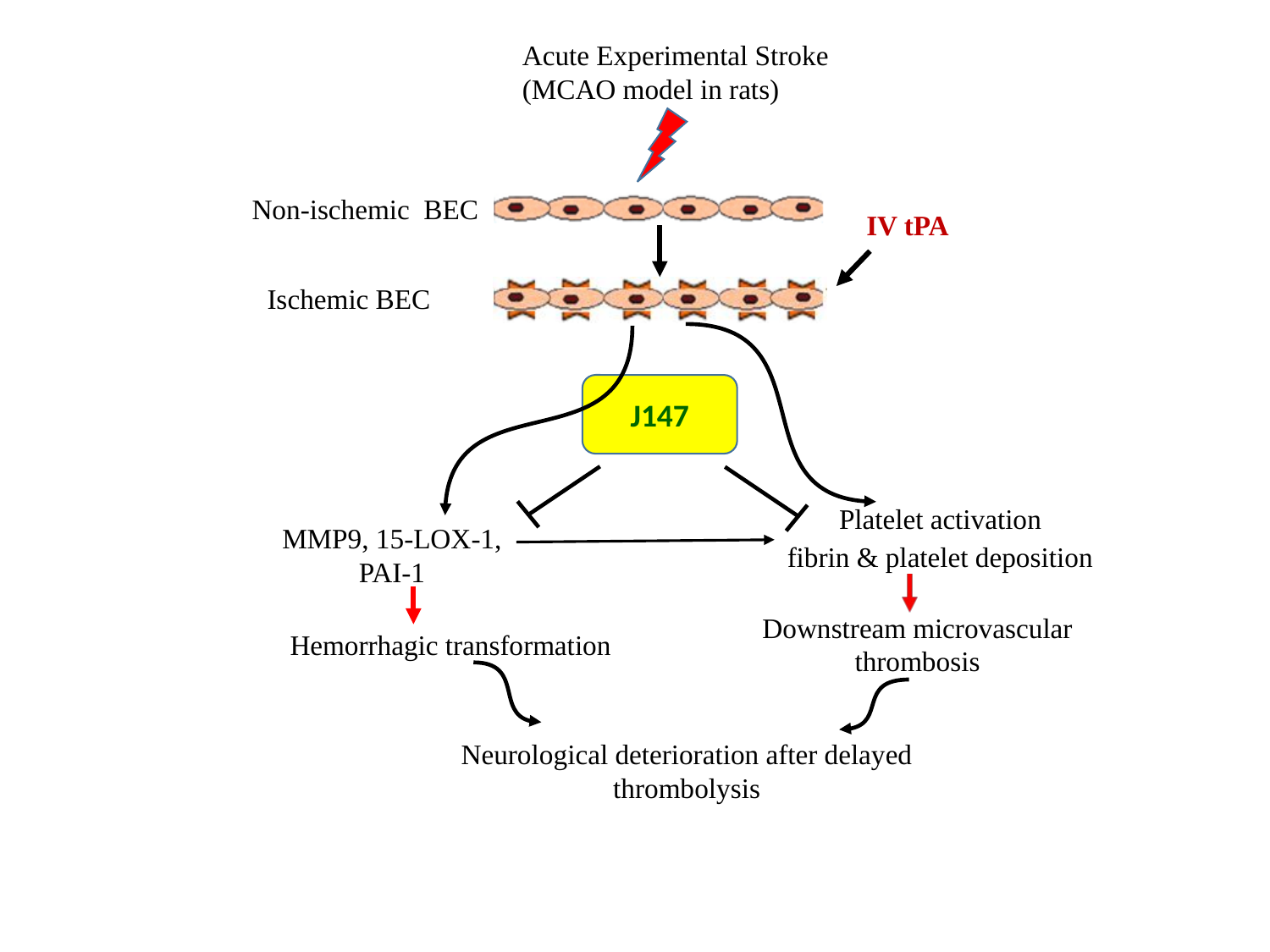

Acute Experimental Stroke
(MCAO model in rats)
Non-ischemic BEC
IV tPA
Ischemic BEC
J147
Platelet activation
fibrin & platelet deposition
MMP9, 15-LOX-1, PAI-1
Downstream microvascular thrombosis
Hemorrhagic transformation
Neurological deterioration after delayed thrombolysis

## Slide 2
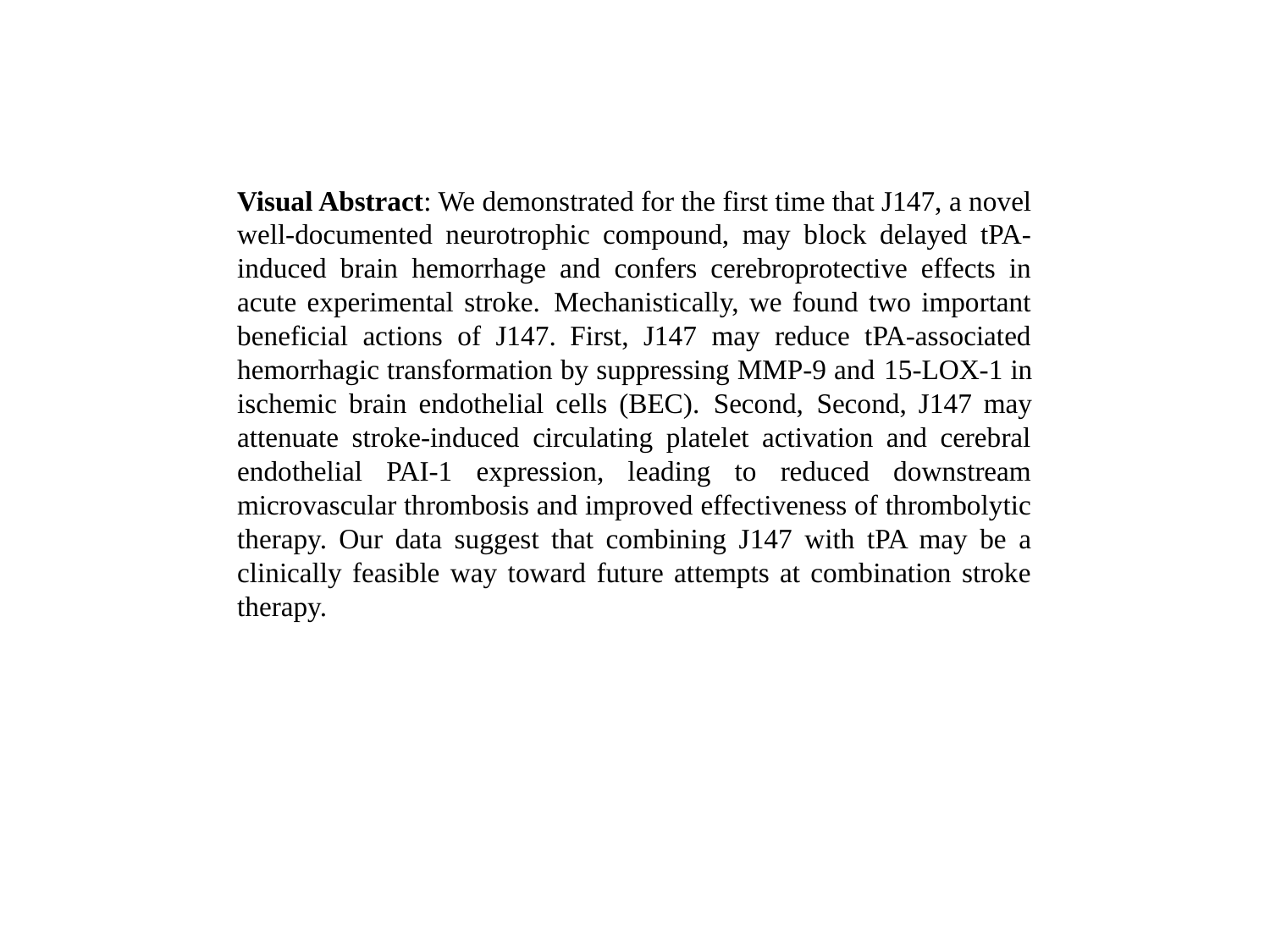

Visual Abstract: We demonstrated for the first time that J147, a novel well‐documented neurotrophic compound, may block delayed tPA-induced brain hemorrhage and confers cerebroprotective effects in acute experimental stroke.  Mechanistically, we found two important beneficial actions of J147.  First, J147 may reduce tPA‐associated hemorrhagic transformation by suppressing MMP‐9 and 15-LOX-1 in ischemic brain endothelial cells (BEC).  Second, Second, J147 may attenuate stroke-induced circulating platelet activation and cerebral endothelial PAI-1 expression, leading to reduced downstream microvascular thrombosis and improved effectiveness of thrombolytic therapy. Our data suggest that combining J147 with tPA may be a clinically feasible way toward future attempts at combination stroke therapy.
