# Supplementary material for: J147 Reduces tPA-Induced Brain Hemorrhage in Acute Experimental Stroke in Rats
Source: Front Neurol. 2022 Mar 2;13:821082. doi: 10.3389/fneur.2022.821082 (PMC8925862; doi:10.3389/fneur.2022.821082)
Supplement: Supplementary file 2 [file Data_Sheet_1.docx]

**SUPPLEMENTAL MATERIAL.**

**I. Supplemental Methods**

**Animals**

Animal experiments were performed in accordance with the National Institutes of Health Guide for the Care and Use of Laboratory Animals and the animal protocols were approved by the Institutional Animal Care and Use Committee at Penn State University College of Medicine. Adult male Wistar rats (weighing 280 to 320g, purchased from Charles River Laboratories, Wilmington, MA) were used.

**Suture transient MCAO and Embolic MCAO Models**

Rats were subjected to suture transient or embolic middle cerebral artery occlusion (MCAO) according to the standard operating procedure (Jin et al., 2014). For the suture transient MCAO model (tMCAO), animals underwent 2 hours of ischemic stroke using silicone rubber-coated monofilaments (503956PK5RE, Doccol, USA). For embolic MCAO Models (eMCAO), a single 4 cm fibrin rich clot was placed in the origin of the right MCA via a modified PE-50 tube (0.3-mm outer diameter). Regional cerebral blood flow (rCBF) in the MCA territory (2 mm posterior and 5 mm lateral to the bregma on the right parietal skull) was monitored with Laser Doppler flowmetry (MSP300XP; ADInstruments Inc). Only animals that showed sustained ischemia to less than 25% rCBF of preischemic baselines were included in this study. Animals with spontaneous recanalization before tPA thrombolysis were excluded. In addition, animals were also excluded from the analysis when the following occurred: death because of surgery or anesthesia that occurred mostly within the first 24 hours after ischemia onset and subarachnoid hemorrhage on postmortem examination.

**Experimental Protocols**

To test the dose-response of J147, animals, subjected to tMCAO, were randomly assigned into the following 4 groups: vehicle [DMSO/PEG200/Saline (5/70/25%)] and varied doses of J147 including 1 mg/kg, 10 mg/kg, and 30 mg/kg. J147 or equal volume of vehicle was administrated via femoral vein at 2 hours after ischemia onset using a syringe infusion pump for 2 min. Rats were euthanized at 72 hours after stroke. Based on the dose–response assessment, the optimal dose of J147 (10 mg/kg) was used in following studies. eMCAO rats were randomly assigned to the following 3 groups: combination treatment with vehicle plus saline (indicated as saline group), vehicle plus rtPA (10mg/kg) (as rtPA group), and J147 plus tPA (as combination therapy group) at 4h after the onset of ischemia. All animals were euthanized at 3 days after stroke with CO_2_. The modified Bederson score, used to determine global neurological function, was performed by a blinded investigator before and at 72 hours after stroke, as we described previously (Jin et al., 2019a; Jin et al., 2018).

**Infarct Volume and Intracerebral Hemorrhage**

At 72 hours after stroke, animals were euthanized with CO_2_. The infarct volume was measured in TTC-stained coronal sections. Then, the TTC-stained sections were homogenized and the hemoglobin levels were assessed by a spectrophotometric assay using Drabkin reagent (Sigma-Aldrich), as we previously described (Jin et al., 2019a; Jin et al., 2018).

**Immunohistochemistry**

At 24 hours after stroke, animals were euthanized with CO2 and transcardially perfused with 200ml ice-cold PBS (0.01 M, pH 7.4) followed by 4% paraformaldehyde (PFA). The brains were removed and post-fixed in 4% PFA solution for overnight at 4°C. Brain samples were then cryoprotected by immersion in 10%, 20%, and 30% gradient sucrose solutions for 48 h at 4°C respectively. After that, the brains were embedded in optimal cutting temperature (OCT) compound. Coronal sections (15um-thick) were cut at 0.4 to 1.4 mm posterior to bregma using a cryostat and mounted on positively charged slides. Every 10th coronal section for a total five sections was used for immunohistochemistry. The primary antibodies: Iba-1 (1:200, Wako), MPO (1:200, Abcam), MMP9 (1:100, R&D), EBA (endothelial barrier antigen, a marker for endothelium in the vessels, 1:500, Covance), fibrinogen (1:200, Dako), thrombocyte (1:200, lifespan) and PAI-1 (1:200, [Novus Biologicals](https://www.novusbio.com/products/serpin-e1-pai-1-antibody_nbp1-19773)) were used. Isotype matched antibodies were used as negative controls. Immunostaining was performed as we described previously.(Jin et al., 2019b) For quantification, the number of immunoreactive vessels were counted in the ischemic boundary zone. All immunostaining data was analyzed by an investigator blinded to experimental groups using Image Pro plus software (version 5.1, Media Cybernetics, Inc.) and data are presented as the density of immunoreactive vessels relative to the imaged area (mm^2^) as described (Jin et al., 2018; Jin et al., 2017).

**Gelatin Zymography and Western blot**

Two assays were performed as described previously (Jin et al., 2017; Lakhan et al., 2013). Protein extracts were obtained from the cerebral cortices. MMP-9 activity in brain homogenates was determined by gelatin zymography. Briefly, brains were removed quickly and dissected on ice, and the ipsilateral and contralateral cortices (between coronal levels +1 mm to −2 mm relative to bregma) were homogenized with lysis buffer (50 mmol/L Tris-HCl [pH 7.4], 150 mmol/L NaCl, 5 mmol/L CaCl_2_, 0.05% Brij-35, and 1% Triton X-100) using Bead Ruptor 24 under the following conditions: 5.65m/s, two times 30 sec with 15 sec pause between. Homogenates were centrifuged at 12,000g for 10 min at 4°C. The supernatant fractions were collected and the protein concentrations were determined. Then, supernatants containing same total proteins were incubated with gelatin-Sepharose 4B (GE Healthcare). After that, the gelatinases were eluted with lysis buffer plus 10% DMSO. Equal amounts of elusion were loaded per lane and separated on 10% SDS-PAGE gels with 0.1% gelatin as substrate. A protein ladder was used to estimate the molecular weight of proteins. Images of the gels were scanned, and quantification was analyzed with Multi-Analyst 1.0 software (Bio-Rad Laboratories). For western blot, brain samples were homogenized in cold RIPA buffer containing protease inhibitor cocktail. Protein samples (40 μg protein each lane) were separated by SDS-PAGE and transferred to polyvinylidene difluoride membrane. The loading control was determined by ponceau S staining. Anti-15 Lipoxygenase 1 (ab244205, 1:1000) was used. Immunopositive bands of horseradish peroxidase (HRP)-conjugated secondary antibodies were detected with an ECL system (GE Healthcare).

**Flow cytometric analysis of platelet activation and platelet-leukocyte aggregation**

Blood was drawn into a heparinized capillary tube via the retro-orbital plexus at 24 h after stroke. Whole blood was diluted 1:10 with Flow Cytometry Staining Buffer (eBioscience) containing rat Fc block anti-rat CD32. For evaluation of platelet activation, sample was co-stained with the FITC mouse anti-rat CD42d (1:200, RPM.4) for platelets and PE/Cy7 mouse anti-rat CD62P (1:100, RMP-1). For detection of platelet–leukocyte aggregates (PLA) formation, samples were co-stained with FITC mouse anti-rat CD42d (1:200, RPM.4), PE mouse anti-rat granulocytes (1:100, RP-1), APC mouse anti-rat CD11b (1:50, WT.5) and PE/Cy7 mouse anti-rat CD45 (1:100, OX-1). Isotype-matched control antibodies were used to differentiate non-specific background signal from specific antibody signals. The samples were analyzed on the BD Accuri™ C6 flow cytometer, as we previously described (Jin et al., 2019a).

**Pharmacokinetic study**

*Sample preparation*: The concentration levels of J147 in rat plasma and brain were evaluated using HPLC-MS/MS method, and dexamethasone (50 ng/mL final concentration) was used as an internal standard. Normal rats received a single IV dose 10 mg/kg J147. Whole blood and brain samples were collected at indicated time points. Whole blood was immediately centrifuged at 2000g for 15 min to collect plasma and stored at -80°C until analysis.  The brain tissue were homogenized in PBS and centrifuged at 10,000 rpm for 10 min to collect the supernatant, and then stored at −80°C until analysis. The calibration standards were prepared by adding 5 μL of J147 working solution into 50 μL blank rat plasma to give the final concentration of J147 at 0.5, 1, 2, 5, 10, 50, 100, 500, 1000 ng/mL. The QC samples were prepared by diluting IV J147 solution [5 mg/ml in DMSO/PEG200/Saline (5/70/25%)] 10000 fold with 50% acetonitrile in water. After that, 5  μL of QC samples were added to 50 μL blank rat plasma. 55 μL standards and 55 μL QC samples were added to 200 μL of acetonitrile for precipitating protein respectively. Then the samples were vortexed for 30 s. After centrifugation 3900 rpm for 15 min at 4 ^o^C, the supernatant was diluted 3 times with water containing 0.1% formic acid. 2 µL of the diluted supernatant was injected into the LC/MS/MS system for quantitative analysis. For unknown study samples, 50 μL sample (plasma or brain homogenate) was mixed with 5 μL blank solution to reach 55 μL total, which is identical to the standard curve samples. Samples were further treated according to calibrating preparation procedure.

*HPLC-MS/MS analysis of J147*: J147 in plasma and brain homogenate was analyzed using an AB API 5500 mass spectrometry coupled with Shimadzu HPLC separation system. Phenomenex Kinetex 2.6µ C18 100A (30*2.1 mm) was used to separate J147 with other isomers as well as impurities. The gradient elution was conducted using a flow rate of 0.6 mL/min with the following conditions for the PO study: initial at 20 % mobile phase B (0.1 % formic acid in 95 % acetonitrile / 5 % water) and 80 % mobile phase A (0.1 % formic acid in 95 % water / 5 % acetonitrile) and hold for 0.3 min, followed by a linear gradient to 100 % mobile phase B in 1.8 min and kept for 0.3 min before going back to initial conditions. For IV study: initial at 25 % mobile phase B (0.1 % formic acid in 95 % acetonitrile / 5 % water) and 75 % mobile phase A (0.1 % formic acid in 95 % water / 5 % acetonitrile) and hold for 0.3 min, followed by a linear gradient to 100 % mobile phase B in 1.8 min and kept for 0.3 min before going back to initial conditions.

The J147 detection was performed on an AB API 5500 mass spectrometer with positive electrospray ionization probe. The compound parameters were as follows: decluster potential (DP) was 106 V for J147 and 100 V for dexamethasone; the entrance potential (EP) was 10 V for J147  and 10 V for dexamethasone; the collision energy (CE) was 25 V for J147 and 11 or 12 V for dexamethasone, while the collision cell exit potential (CXP) was 14 V for J147 and 10 V for dexamethasone. The optimized ion source parameters were set as follows: gas (CUR) was 30 and 40 L/h, the collision gas (CAD) was 10. The ionSpray voltage was 5500 V, the temperature was 550℃ and  500℃, gas 1 was 50 and 60 L/h, and gas 2 was 50 and 60 L/h. The multiple reaction monitoring mode (MRM) was used for quantification with the transitions setting at m/z 351.115/216.000 for J147 and 393.3/373.1 for dexamethasone respectively. All peaks were integrated and quantified using Analyst 1.7.1 and 1.6.2 software.

**Assessment of tissue plasminogen activator inhibition by J147**

Tissue plasminogen activator (tPA) is a serine protease that catalyzes the conversion of plasminogen to plasmin, thereby facilitating the breakdown of blood clots. To determine whether J147 is an inhibitor of tPA, a suitable commercially available inhibition assay was identified. Inhibition was assayed using the Plasminogen Activator Inhibitor-1 Activity Assay Kit from BioVision Inc. (catalog # K2040-100), according to manufacturer's instructions. Plasminogen Activator Inhibitor-1 (PAI-1) is a naturally occurring protein inhibitor of tPA that can be used as a control when determining potential J147 inhibitory activity. The kit is designed for measuring PAI-1 activity, but is adaptable to measuring the activity of other tPA inhibitors.

The assay is a two-step colorimetric assay. In the first step, inhibitor samples are incubated with a known amount of tPA. In the second step, the residual free tPA converts plasminogen to plasmin, which in turn hydrolyzes a plasmin chromogenic substrate thereby releasing the chromophore paranitroanaline (pNA, OD 405 nm). The absorbance of the released pNA is directly proportional to the tPA activity.

The assay was performed by SAMDI Tech Inc. (Chicago, Ill.) and was carried out according to the instructions provided with the kit. J147 was prepared as a 10 mM stock in DMSO immediately before performing the assay. A 4-fold dilution series of the stock was prepared in DMSO and added to the reactions at the appropriate volumes to give the desired final concentrations. Under all conditions tested, the final concentration of DMSO in the assay was 1% or less. The positive control was 40 U/ml of tPA, without J147 or PAI-1. The negative control was 40 U/ml of tPA, without plasminogen enzyme. Reactions were performed in duplicate.

PAI-1 was obtained from BioVision (catalog # 6377-100). A standard curve for PAI-1 was generated according to the instructions provided with the assay kit. This standard curve, shown below, was generated by preparing a dilution series of the tissue plasminogen activator protein (tPA), and shows the expected signal at any given concentration of PAI-1 protein. In this plot, the X-axis is also equivalent to the inverse of tPA concentration. The standard curve is well-behaved and linear, as expected.

**Supplemental References**

Jin, R., Xiao, A.Y., Chen, R., Granger, D.N., Li, G., 2017. Inhibition of CD147 (cluster of differentiation 147) ameliorates acute ischemic stroke in mice by reducing thromboinflammation. Stroke 48, 3356–3365.

Jin, R., Xiao, A.Y., Li, J., Wang, M., Li, G., 2019a. PI3Kγ (Phosphoinositide 3-Kinase-γ) Inhibition Attenuates Tissue-Type Plasminogen Activator-Induced Brain Hemorrhage and Improves Microvascular Patency after Embolic Stroke. Hypertension. https://doi.org/10.1161/HYPERTENSIONAHA.118.12001

Jin, R., Xiao, A.Y., Li, J., Wang, M., Li, G., 2019b. PI3Kγ (Phosphoinositide 3-Kinase-γ) Inhibition Attenuates Tissue-Type Plasminogen Activator-Induced Brain Hemorrhage and Improves Microvascular Patency After Embolic Stroke. Hypertens (Dallas, Tex 1979). https://doi.org/10.1161/HYPERTENSIONAHA.118.12001

Jin, R., Xiao, A.Y., Liu, S., Wang, M., Li, G., 2018. Taurine reduces tPA (Tissue-Type plasminogen activator)-induced hemorrhage and microvascular thrombosis after embolic stroke in rat. Stroke 49, 1708–1718.

Jin, R., Zhu, X., Li, G., 2014. Embolic middle cerebral artery occlusion (MCAO) for ischemic stroke with homologous blood clots in rats. J Vis Exp. https://doi.org/10.3791/51956

Lakhan, S.E., Kirchgessner, A., Tepper, D., Leonard, A., 2013. Matrix metalloproteinases and blood-brain barrier disruption in acute ischemic stroke. Front Neurol. https://doi.org/10.3389/fneur.2013.00032

**II. Supplemental Data**

To ensure the assay performed as expected, a control IC50 was generated using the PAI-1 protein. A 12-point, 3-fold dilution series of PAI-1 was generated in assay buffer covering a concentration range of 5.5 µM to 0.03 nM. Percent inhibition was calculated using the included positive and negative controls. For the PAI-1 protein, an IC50 value of 0.27 nM was obtained. A plot of the results of the PAI-1 IC50 determination is shown below.

J147 inhibition was tested under two conditions:

Condition 1: J147 was incubated with tPA for 20 minutes at room temperature and 20 minutes at 37°C before performing the activity assay in order to replicate the original PAI-1 assay conditions.

Condition 2: J147 was introduced immediately before initiating the 90-minute plasminogen detection reaction.

For both conditions, the compound volumes were adjusted so that the compound concentrations in the final reactions were the same, covering the range of 10 µM to 10 nM.

As shown in the following graph, the presence of J147 had no effect on tPA activity under either condition, indicating J147 does not inhibit tPA serine protease activity against plasminogen under these conditions.

|  | Group | Treatment | Sample size Survived No. (total animals) |
| --- | --- | --- | --- |
| Dose-response experiments (3 days) | tMCAO + Vehicle | Vehicle (IV at 2h) | 9 (11) |
|  | tMCAO + J147 (1 mg/kg) | J147 (IV at 2h) | 9 (10) |
|  | tMCAO + J147 (10 mg/kg) | J147 (IV at 2h) | 9 (11) |
|  | tMCAO + J147 (30 mg/kg) | J147 (IV at 2h) | 9 (9) |
|  |  |  |  |
|  | eMCAO + Vehicle | Vehicle (IV at 2h) | 6 (8) |
|  | eMCAO + J147 (1 mg/kg) | J147 (IV at 2h) | 6 (9) |
|  | eMCAO + J147 (10 mg/kg) | J147 (IV at 2h) | 6 (7) |
|  | eMCAO + J147 (30 mg/kg) | J147 (IV at 2h) | 6 (8) |
| Effects of combination therapy (3 days) | eMCAO + Vehicle | Vehicle + saline (IV at 4h) | 9 (12) |
|  | eMCAO + tPA | Vehicle + tPA (IV at 4h) | 15 (22) |
|  | eMCAO + J147 + tPA | J147 + tPA (IV at 4h) | 15 (17) |
| IHC studies (24 hours) | Sham | No treatment | 5 (5) |
|  | eMCAO + Vehicle | Vehicle + saline (IV at 4h) | 5 (6) |
|  | eMCAO + tPA | Vehicle + tPA (IV at 4h) | 5 (6) |
|  | eMCAO + J147 + tPA | J147 + tPA ( IV at 4h) | 5 (6) |
| Zymography and Western blot studies  (24 hours) | Sham | No treatment | 5 (5) |
|  | eMCAO + Vehicle | Vehicle + saline (IV at 4h) | 5 (6) |
|  | eMCAO + tPA | Vehicle + tPA (IV at 4h) | 5 (6) |
|  | eMCAO + J147 + tPA | J147 + tPA ( IV at 4h) | 5 (6) |
| Flow cytometry studies  (24 hours) | Sham | No treatment | 5 (5) |
|  | eMCAO + Vehicle | Vehicle + saline (IV at 4h) | 5 (6) |
|  | eMCAO + tPA | Vehicle + tPA (IV at 4h) | 5 (6) |
|  | eMCAO + J147 + tPA | J147 + tPA ( IV at 4h) | 5 (6) |
| Pharmacokinetics study | Normal animal +J147 | J147 (IV) | 15 (15) |
| IV, intravenously. | | | |

**Suppl. Table II. Summary of experimental groups, treatments, and sample sizes**

**Suppl. Table III. J147 pharmacokinetic parameters**

|  | Cl_obs_ (mL/min/kg) |  | t_1/2_ (h) | C_0_ (ng/mL) | AUC_last_ (h*ng/mL) | AUC_Inf_ (h*ng/mL) | AUC_Extrap, obs_ (%) | MRT_Inf_ (h) | AUC_last_/D (h*mg/mL) | V_ss, obs_ (L/kg) |
| --- | --- | --- | --- | --- | --- | --- | --- | --- | --- | --- |
| Mean | 94 |  | 6.33 | 6452 | 1769 | 1789 | 1.07 | 1.72 | 177 | 9.7 |
| SD | 11 |  | 1.15 | 2234 | 206 | 213 | 0.30 | 0.13 | 21 | 1.3 |

Cl_obs_ = total body clearance of the drug from plasma, t_1/2_ = elimination half-life, C_0_ = plasma drug concentration at time zero following intravenous injection, AUC_last_ = area under the plasma concentration-time curve from time zero to time of last measurable concentration, AUC_inf_ = area under the plasma concentration-time curve from time zero to infinity, AUC_Extrap_obs_ = Area under the plasma concentration-time curve extrapolated from time t to infinity as a percentage of total AUC, MRT_inf_ = mean residence time, D = dose, V_ss_obs_ = apparent volume of distribution at steady state
